# Combined effect of physical activity and leisure time sitting on long-term risk of incident obesity and metabolic risk factor clustering

**DOI:** 10.1007/s00125-014-3323-8

**Published:** 2014-07-31

**Authors:** Joshua A. Bell, Mark Hamer, G. David Batty, Archana Singh-Manoux, Séverine Sabia, Mika Kivimaki

**Affiliations:** 1 Department of Epidemiology and Public Health, University College London, 1-19 Torrington Place, London, WC1E 6BT UK; 2 INSERM, U1018, Centre for Research in Epidemiology and Population Health, Villejuif, France

**Keywords:** Epidemiology, Exercise, Metabolic syndrome, Obesity, Weight regulation

## Abstract

**Aims/hypothesis:**

Our study aimed to investigate the combined effects of moderate-to-vigorous physical activity and leisure time sitting on the long-term risk of obesity and clustering of metabolic risk factors.

**Methods:**

The duration of moderate and vigorous physical activity and of leisure time sitting was assessed by questionnaire between 1997 and 1999 among 3,670 participants from the Whitehall II cohort study (73% male; mean age 56 years). Multivariable-adjusted logistic regression models examined associations of physical activity and leisure time sitting tertiles with odds of incident obesity (BMI ≥ 30 kg/m^2^) and incident metabolic risk factor clustering (two or more of the following: low HDL-cholesterol, high triacylglycerol, hypertension, hyperglycaemia, insulin resistance) at 5 and 10 year follow-ups.

**Results:**

Physical activity, but not leisure time sitting, was associated with incident obesity. The lowest odds of incident obesity after 5 years were observed for individuals reporting both high physical activity and low leisure time sitting (OR = 0.26; 95% CI 0.11, 0.64), with weaker effects after 10 years. Compared with individuals in the low physical activity/high leisure time sitting group, those with intermediate levels of both physical activity and leisure time sitting had lower odds of incident metabolic risk factor clustering after 5 years (OR 0.53; 95% CI 0.36, 0.78), with similar odds after 10 years.

**Conclusions/interpretation:**

Both high levels of physical activity and low levels of leisure time sitting may be required to substantially reduce the risk of obesity. Associations with developing metabolic risk factor clustering were less clear.

**Electronic supplementary material:**

The online version of this article (doi:10.1007/s00125-014-3323-8) contains peer-reviewed but unedited supplementary material, which is available to authorised users.

## Introduction

Obesity is associated with a clustering of metabolic abnormalities, such as hypertension, dyslipidaemia, insulin resistance and hyperglycaemia [1]. In general populations, however, metabolic risk factor clustering has been observed in both obese and non-obese adults [2, 3]. The magnitude of long-term cardiovascular disease and mortality risk has been shown to depend on whether these factors are comorbid [4]. This suggests that obesity and metabolic risk factor clustering may be partly distinct clinical components of overall metabolic health.

Physical activity and sedentariness are two common lifestyle-related behaviours associated with both obesity and metabolic health, as well as with chronic diseases such as type 2 diabetes and cardiovascular disease, and with all-cause mortality [5–8]. Physical activity is assessed as movements of a light, moderate or vigorous intensity, requiring a metabolic equivalent (MET) value of at least 1.6 units. Sedentary behaviour, or ‘sitting’, in turn refers to the absence of movement, requiring ≤ 1.5 METs [9], and may influence health independently through mechanisms such as dysregulation of lipoprotein lipase activity and inflammation [6, 10], or simply through displacement of moderate-to-vigorous physical activity [11].

There is evidence, although mostly cross-sectional, that physical activity is more strongly associated with obesity than is sitting [12, 13] and that these behaviours are associated with metabolic risk factors including blood pressure, plasma lipids, blood glucose and insulin [6, 14–17]. However, there is limited prospective evidence to support their role as protective factors against future weight gain and obesity [18–20]. Physical activity has been associated with a reduced risk of developing metabolic risk factor clustering [21, 22], while sitting has been associated with an increased risk [23, 24].

Studies often consider physical activity and sitting individually; however, these behaviours are closely linked, with modern-day inactive lifestyles being characterised by both insufficient physical activity and excessive leisure time sitting [25]. Evidence on their combined effects is limited and it is difficult to infer the causal direction of effects on overweight [26] or obesity [27, 28] from cross-sectional studies. One prospective study found that the combination of high physical activity and low sitting is associated with a lower risk of developing obesity [29]. Their combined effect on the risk of developing metabolic risk factor clustering, however, has not been investigated. Furthermore, the relative importance of physical activity and leisure time sitting combinations in relation to the development of obesity and metabolic risk factor clustering has not been studied within a single analytical framework.

This study aimed to clarify these associations by prospectively investigating the long-term risk of incident obesity and incident metabolic risk factor clustering among adults with different levels and combinations of physical activity and leisure time sitting.

## Methods

### Study population

The Whitehall II study is an occupational cohort study of British civil servants (government employees) in which 10,308 men and women were recruited between 1985 and 1988 [30]. Participants have been followed up every 5 years with clinical examinations. Data on sitting time were first recorded in 1997–1999, which served as the baseline for this study. Participants provided written informed consent. Ethical approval was obtained from the University College London Research Ethics Committee.

### Physical activity and leisure time sitting

As part of the 1997–1999 questionnaire, physical activity was assessed using a modified 20-item version of the validated Minnesota Leisure Time Physical Activity Questionnaire [31–33]. Participants reported the frequency and duration of various activities including sports, walking, cycling, home maintenance and gardening. Duration of moderate-to-vigorous physical activity (≥3 METs) was used in analyses and classified into tertiles (low, 0–1.50 h/week; intermediate, 1.56–4.25 h/week; high, 4.27–20.56 h/week). Participants were also asked: ‘On average, how many hours a week do you spend sitting at home, e.g. watching TV, sewing, at a desk?’, for which participants selected one of eight responses: none, 1, 2–5, 6–10, 11–20, 21–30, 31–40, 40+ h. The midpoint for each time slot was summed to form a continuous scale, with each unit representing a 1 h change in sitting time. Total leisure time sitting was then divided into tertiles (low, 0–11.5 h/week; intermediate, 15–23 h/week; high, 25–90 h/week). Tertiles of physical activity and leisure time sitting were further combined to form nine groups. ‘Low activity/high sitting’ was used as the reference group, representing the assumed lowest level of energy expenditure and, thus, the least favourable combination.

### Incident obesity

Objectively measured anthropometrics were assessed in 1997–1999 (baseline), 2002–2004 (5 year follow-up) and 2007–2009 (10 year follow-up) and used to compute BMI using the standard formula: weight in kilograms divided by the square of height in metres. Obesity was defined as BMI ≥ 30 kg/m^2^ (with ‘non-obese’ defined as BMI < 30 kg/m^2^).

### Incident metabolic risk factor clustering

Objectively measured metabolic risk factors were assessed in 1997–1999 (baseline), 2002–2004 (5 year follow-up) and 2007–2009 (10 year follow-up) and used to define metabolic health status based on comprehensive criteria [2]. ‘Metabolic risk factor clustering’ was defined as having two or more of the following risk factors: HDL-cholesterol < 1.03 mmol/l for men and < 1.29 mmol/l for women; blood pressure ≥ 130/85 mmHg or taking antihypertensive medication; fasting plasma glucose ≥ 5.6 mmol/l or taking diabetic medication; triacylglycerol ≥ 1.7 mmol/l; HOMA of insulin resistance (HOMA-IR) > 3.20 (90th percentile value in 1997–1999).

### Covariates

Covariates used in the present analyses were assessed in 1997–1999 and included age, sex and ethnicity (‘non-white’, ‘white’), socioeconomic status as indicated by British civil service occupational position (‘administrative’, ‘professional/executive’, ‘clerical/support’), health status as represented by the self-reported presence of a long-standing illness (‘yes’, ‘no’), and health behaviours as indicated by frequency of fruit and vegetable consumption (‘at least one serving per day’; ‘less than one serving per day’), cigarette smoking status (‘never smoker’, ‘ex-smoker’, ‘current smoker’) and units of alcohol consumed in the previous week.

### Statistical analyses

Logistic regression models were used to compute ORs with accompanying 95% CIs as estimates of associations of physical activity and leisure time sitting, separately and in combination, with incident obesity and incident metabolic risk factor clustering at 5 and 10 years of follow-up. The 10 year follow-up assessed the cumulative incidence for each outcome, considering changes at 5 or 10 years. Participants who were obese at baseline were excluded from analyses for incident obesity. Likewise, participants with metabolic risk factor clustering at baseline were excluded from analyses for incident metabolic risk factor clustering. Estimates were first adjusted for age, sex and ethnicity (the minimally adjusted model), and then further adjusted for occupational position, health status and health behaviours (the multivariable-adjusted model). If data were unavailable for health behaviour covariates at the 1997–1999 assessment, data from 1991–1994 were used. Statistical interaction between physical activity and leisure time sitting was tested by including the product term of the corresponding tertiles in relation to each outcome in logistic regression models. Analyses were performed using SPSS software, version 19 (IBM SPSS, Armonk, NY, USA), with two-tailed *p* < 0.05 indicating statistical significance.

### Sensitivity analyses

In order to investigate adverse metabolic change in greater detail, we used linear regression models to further estimate associations of physical activity and leisure time sitting, separately and in combination, with change in the number of metabolic risk factors (including obesity; range 0–6) between baseline and follow-up. The number of risk factors (high blood pressure, high blood glucose, high triacylglycerol, low HDL-cholesterol, insulin resistance and obesity) at baseline was subtracted from the number of risk factors in 2002–2004 to estimate the difference after 5 years. Likewise, the number of risk factors at baseline was subtracted from the number of risk factors in 2007–2009 to estimate 10 year changes. A positive value indicated an increase in the number of risk factors over time, whereas a negative value indicated a decrease in the number of risk factors over time.

## Results

### Sample characteristics

The study sample (defined as the group of participants with data on physical activity and leisure time sitting at baseline, and obesity status, metabolic risk factors and covariates at baseline and 5 years of follow-up) comprised 3,670 individuals. This group was approximately three-quarters male, with a mean age of 55.5 (SD 6.0) years at baseline. Compared with individuals included in analyses, those excluded were more likely to be women (36.2% vs 27.5%; *p* < 0.001), of a non-white ethnicity (13.1% vs 7.0%; *p* < 0.001) and from the lowest occupational position group (18.3% vs 11.0%; *p* < 0.001).

As shown in Table 1, 17.6% of the low physical activity group was considered obese, compared with 11.4% of those in the high physical activity group (*p* < 0.05). The baseline prevalence of obesity was similar in the low (12.1%) and high leisure time sitting groups (14.4%; *p* > 0.05). Nearly 36% of individuals in the low physical activity group had metabolic risk factor clustering at baseline, compared with approximately 28% in the high physical activity group (*p* < 0.05). Conversely, nearly 34% of the high leisure time sitting group had metabolic risk factor clustering, compared with about 30% of those in the low leisure time sitting group (*p* > 0.05).

**Table 1:** Characteristics of participants in the Whitehall II cohort study by level of physical activity and leisure time sitting (*N* = 3,670)

Characteristic	Physical activity level			Leisure time sitting level		
	Low (*n* = 1,205)	Intermediate (*n* = 1,254)	High (*n* = 1,211)	High (*n* = 1,336)	Intermediate (*n* = 1,311)	Low (*n* = 1,023)
Baseline						
Male, *n* (%)	706 (58.6)	917 (73.1)*	1,037 (85.6)*	995 (74.5)	955 (72.8)	710 (69.4)^†^
Age, years	54.8 (5.7)	55.0 (5.9)	56.7 (6.0)*	56.6 (6.0)	54.8 (5.9)^†^	54.9 (5.7)^†^
Non-white ethnicity, *n* (%)	154 (12.8)	66 (5.3)*	36 (3.0)*	75 (5.6)	69 (5.3)	112 (10.9)^†^
Lowest employment grade, *n* (%)	244 (20.2)	114 (9.1)*	46 (3.8)*	134 (10)	118 (9.0)	152 (14.9)^†^
Systolic blood pressure, mmHg	122.5 (16.5)	122.0 (15.9)	123.5 (16.0)	123.2 (15.9)	122.3 (16.4)	122.5 (16.1)
Diastolic blood pressure, mmHg	77.3 (10.3)	77.5 (10.5)	77.6 (10.4)	77.7 (10.2)	77.3 (10.6)	77.4 (10.5)
HDL-cholesterol, mmol/l	1.5 (0.4)	1.5 (0.4)	1.5 (0.4)	1.4 (0.4)	1.5 (0.4)	1.5 (0.4)
Fasting glucose, mmol/l	5.2 (1.1)	5.1 (0.8)	5.2 (1.0)	5.2 (0.9)	5.2 (0.9)	5.2 (1.2)
HOMA-IR	2.7 (6.3)	2.1 (2.5)*	2.1 (3.6)*	2.4 (5.5)	2.2 (2.8)	2.3 (4.5)
Triacylglycerol, mmol/l	1.4 (0.9)	1.3 (0.9)*	1.3 (0.8)*	1.4 (0.9)	1.3 (0.8)^†^	1.3 (0.8)^†^
At least one fruit/vegetable serving per day, *n* (%)	815 (67.6)	956 (76.2)*	963 (79.5)*	1,011 (75.7)	986 (75.2)	737 (72.0)^†^
Current smoker, *n* (%)	137 (11.4)	103 (8.2)*	69 (5.7)*	130 (9.7)	103 (7.9)	76 (7.4)
Alcohol units in previous week	12.4 (15.9)	13.7 (14.6)*	15.5 (14.6)*	14.6 (16.0)	14.1 (13.9)	12.8 (15.3)^†^
Obese, *n* (%)	212 (17.6)	160 (12.8)*	138 (11.4)*	193 (14.4)	193 (14.7)	124 (12.1)
Metabolic risk factor clustering, *n* (%)	429 (35.6)	377 (30.1)*	335 (27.7)*	447 (33.5)	389 (29.7)^†^	305 (29.8)
Adverse metabolic changes at follow-up						
Incident obesity after 5 years, *n* (%)^a^	79 (8.0)	63 (5.8)*	52 (4.8)*	74 (6.5)	60 (5.4)	60 (6.7)
Incident obesity after 10 years, *n* (%)^b^	101 (11.9)	72 (7.6)*	68 (6.9)*	84 (8.4)	83 (8.3)	74 (9.5)
Incident metabolic risk factor clustering after 5 years, *n* (%)^c^	183 (23.6)	170 (19.4)*	198 (22.6)	223 (25.1)	182 (19.7)^†^	146 (20.3)^†^
Incident metabolic risk factor clustering after 10 years, *n* (%)^d^	229 (33.6)	217 (28.2)*	252 (31.3)	274 (34.8)	238 (28.5)^†^	186 (29.4)^†^

Levels are based on tertiles; values are mean (SD) unless otherwise noted

^a^Sample size = 3,160

^b^Sample size = 2,778

^c^Sample size = 2,529

^d^Sample size = 2,254

*Significantly different from low physical activity group (*p* < 0.05)

^†^Significantly different from high leisure time sitting group (*p* < 0.05)

### Incident obesity

As shown in Table 1, the rate of incident obesity was lower in the high compared with the low physical activity group after 5 years (4.8% vs 8.0%; *p* < 0.05) and after 10 years (6.9% vs 11.9%; *p* < 0.05). Incident obesity did not differ by level of leisure time sitting at either follow-up (*p* > 0.05). Compared with having a low level of physical activity, having a high level was associated with 0.64 (95% CI 0.44, 0.93) times lower odds of incident obesity after 5 years, with 0.63 (95% CI 0.45, 0.88) times lower odds after 10 years in models adjusted for age, sex and ethnicity (electronic supplementary material [ESM] Table 1). In multivariable-adjusted models (Table 2), lower odds of incident obesity were observed with increasing levels of physical activity independently of leisure time sitting after 10 years only (*p* for trend = 0.02). Compared with being in the low physical activity group, being in the intermediate and the high group was associated with 0.66 (95% CI 0.47, 0.91) and 0.67 (95% CI 0.48, 0.95) times lower odds of incident obesity after 10 years, respectively. Leisure time sitting level was not associated with incident obesity at either follow-up.

**Table 2:** Separate associations of physical activity and leisure time sitting level at baseline with incident obesity and incident metabolic risk factor clustering at follow-up

	Incident obesity OR (95% CI)		Incident metabolic risk factor clustering OR (95% CI)	
	After 5 years (*n* = 3,160)	After 10 years (*n* = 2,778)	After 5 years (*n* = 2,529)	After 10 years (*n* = 2,254)
Physical activity level				
Low	1.00 (reference)	1.00 (reference)	1.00 (reference)	1.00 (reference)
Intermediate	0.76 (0.53, 1.08)	0.66 (0.47, 0.91)	0.77 (0.60, 0.98)	0.76 (0.60, 0.95)
High	0.70 (0.47, 1.03)	0.67 (0.48, 0.95)	0.87 (0.68, 1.12)	0.85 (0.67, 1.07)
*p* for trend	0.06	0.02	0.33	0.21
Leisure time sitting level				
High	1.00 (reference)	1.00 (reference)	1.00 (reference)	1.00 (reference)
Intermediate	0.80 (0.56, 1.15)	0.96 (0.69, 1.32)	0.79 (0.63, 0.99)	0.78 (0.63, 0.97)
Low	1.01 (0.71, 1.45)	1.10 (0.79, 1.55)	0.83 (0.65, 1.06)	0.83 (0.65, 1.04)
*p* for trend	0.96	0.64	0.09	0.07

Separate associations are mutually adjusted; models adjusted for age, sex, ethnicity, occupational position, frequency of fruit and vegetable consumption, smoking status, alcohol consumption and the presence of a long-standing illness

Compared with the combination of low physical activity and high leisure time sitting, most groups trended towards lower odds of incident obesity, but the combination of high physical activity and low leisure time sitting was associated with the lowest odds after 5 years (OR 0.23; 95% CI 0.10, 0.57) and after 10 years (OR 0.47; 95% CI 0.24, 0.91) in minimally adjusted models (ESM Table 2). Lower odds of incident obesity were observed for increasing levels of physical activity only within the low leisure time sitting group (*p* for trend = 0.001); and similarly lower odds of incident obesity were observed for decreasing levels of leisure time sitting only within the high physical activity group (*p* for trend = 0.01; *p* for interaction = 0.02 after 5 years). In multivariable-adjusted models (Table 3), only the combination of high physical activity and low leisure time sitting was associated with lower odds of incident obesity after 5 years (OR 0.26; 95% CI 0.11, 0.64). This result was also observed after 10 years, although the effect size was smaller (OR 0.51; 95% CI 0.26, 1.00; *p* = 0.05; *p* for interaction = 0.37).

**Table 3:** Combined associations of physical activity and leisure time sitting level at baseline with incident obesity at follow-up

	Incident obesity Odds ratio (95% CI)							
	After 5 years *n* = 3,160				After 10 years *n* = 2,778			
	Leisure time sitting level				Leisure time sitting level			
Physical activity level	High	Intermediate	Low	*p*-trend	High	Intermediate	Low	*p*-trend
Low	1.00 (reference)	0.64 (0.36, 1.15)	1.11 (0.64, 1.93)	0.85	1.00 (reference)	0.91 (0.55, 1.53)	1.31 (0.78, 2.20)	0.35
Intermediate	0.55 (0.30, 1.02)	0.55 (0.30, 1.00)	0.99 (0.57, 1.71)	0.07	0.60 (0.33, 1.07)	0.65 (0.38, 1.13)	0.84 (0.49, 1.45)	0.36
High	0.81 (0.46, 1.43)	0.72 (0.40, 1.29)	0.26 (0.11, 0.64)	0.02	0.81 (0.48, 1.39)	0.74 (0.43, 1.27)	0.51 (0.26, 1.00)	0.21
*p*-trend	0.43	0.47	0.002		0.39	0.59	0.01	
*p*-interaction		0.02				0.37		

Models adjusted for age, sex, ethnicity, occupational position, frequency of fruit and vegetable consumption, smoking status, alcohol consumption and the presence of a long-standing illness.

### Incident metabolic risk factor clustering

As shown in Table 1, incident metabolic risk factor clustering after 5 or 10 years was less prevalent in the intermediate compared with the low physical activity group (*p* < 0.05), while this was not observed for the high physical activity group (*p* > 0.05). Incident metabolic risk factor clustering was less prevalent in the low compared with the high leisure time sitting group after 5 years (20.3% vs 25.1%; *p* < 0.05) and after 10 years (29.4% vs 34.8%; *p* < 0.05). In models adjusted for age, sex and ethnicity (ESM Table 1), being in the intermediate physical activity group was associated with lower odds of incident metabolic risk factor clustering after 5 years (OR 0.76; 95% CI 0.60, 0.97) and after 10 years (OR 0.75; 95% CI 0.60, 0.94), independently of leisure time sitting. Similarly reduced odds were observed for intermediate levels of physical activity in multivariable-adjusted models (Table 2). Estimates for the high physical activity group were consistently below 1.00, but did not reach statistical significance at conventional levels at either follow-up point. Compared with being in the high leisure time sitting group, being in the intermediate group was associated with lower odds of incident metabolic risk factor clustering after 5 years (OR 0.79; 95% CI 0.63, 0.99) and after 10 years (OR 0.78; 95% CI 0.63, 0.97) in multivariable-adjusted models.

Compared with the combination of low physical activity and high leisure time sitting, most groups trended towards lower odds of incident metabolic risk factor clustering after 5 and 10 years, adjusting for age, sex and ethnicity (ESM Table 2); however, the greatest reduction in odds was observed for the intermediate physical activity/low leisure time sitting combination (OR 0.50; 95% CI 0.33, 0.76) after 5 years and the intermediate physical activity/intermediate leisure time sitting combination after 10 years (OR 0.52; 95% CI 0.36, 0.76). In multivariable-adjusted models (Table 4), the lowest odds of incident metabolic risk factor clustering were observed for the intermediate physical activity/intermediate leisure time sitting combination after 5 years (OR 0.53; 95% CI 0.36, 0.78; *p* for interaction = 0.35) and after 10 years (OR 0.53; 95% CI 0.36, 0.77; *p* for interaction = 0.47).

**Table 4:** Combined associations of physical activity and leisure time sitting level at baseline with incident metabolic risk factor clustering at follow-up

	Incident metabolic risk factor clustering Odds ratio (95% CI)							
	After 5 years *n* = 2,529				After 10 years *n* = 2,254			
	Leisure time sitting level				Leisure time sitting level			
Physical activity level	High	Intermediate	Low	*p*-trend	High	Intermediate	Low	*p*-trend
Low	1.00 (reference)	0.64 (0.43, 0.95)	0.74 (0.48, 1.13)	0.12	1.00 (reference)	0.70 (0.47, 1.02)	0.84 (0.55, 1.27)	0.31
Intermediate	0.75 (0.51, 1.10)	0.53 (0.36, 0.78)	0.54 (0.35, 0.82)	0.11	0.81 (0.56, 1.19)	0.53 (0.36, 0.77)	0.57 (0.38, 0.85)	0.08
High	0.67 (0.45, 0.98)	0.71 (0.49, 1.05)	0.70 (0.46, 1.05)	0.79	0.72 (0.50, 1.04)	0.72 (0.50, 1.05)	0.68 (0.45, 1.02)	0.77
*p*-trend	0.01	0.49	0.45		0.10	0.97	0.82	
*p*-interaction		0.35				0.47		

Models adjusted for age, sex, ethnicity, occupational position, frequency of fruit and vegetable consumption, smoking status, alcohol consumption and the presence of a long-standing illness.

### Sensitivity analyses

Sensitivity analyses examining continuous change in the number of metabolic risk factors (including obesity) suggested that neither high physical activity nor low leisure time sitting were independently associated with change in the number of metabolic risk factors after 5 or 10 years (ESM Tables 3, 4). No combination of physical activity and leisure time sitting was associated with change in the number of metabolic risk factors after 5 or 10 years.

## Discussion

This study prospectively examined the combined effect of moderate-to-vigorous physical activity and leisure time sitting on the long-term risk of two related adverse metabolic changes: obesity and metabolic risk factor clustering. High levels of physical activity were associated with a slightly reduced risk of becoming obese after 10 years of follow-up; however, the combined effect of high physical activity and low leisure time sitting after 5 years was much larger, suggesting a substantially reduced risk of developing obesity for highly active individuals who also engage in low amounts of sitting in their leisure time. No such interaction was observed in relation to incident metabolic risk factor clustering: physical activity and leisure time sitting each showed comparable associations with risk of incident metabolic risk factor clustering, with reduced risk observed for intermediate levels only. These results were robust to adjustment for a wide range of potentially confounding factors, including socioeconomic status, smoking behaviour, alcohol consumption and health status.

The present findings, based on longitudinal comparisons of separate and combined associations of physical activity and leisure time sitting, add to the literature by suggesting that the combination of high physical activity and low leisure time sitting is a stronger protective factor against becoming obese than either behaviour on its own. This finding is in agreement with previous studies. In a recent investigation of young adults, for example, increased physical activity reduced the risk of becoming obese after 5 years only within individuals who also showed lower screen-based sitting time; although this association was observed in females only [29].

The mechanisms underlying this interaction are unclear. In principle, lower levels of leisure time sitting may strengthen protective effects of higher physical activity, either through independent physiological mechanisms [6] or as a marker for greater engagement in low-intensity activity, such as standing [34]. Physical activity and leisure time sitting combinations may also simply represent incremental increases in energy expenditure, with the lowest physical activity/highest leisure time sitting group expending the least amount of energy overall, and the highest physical activity/lowest leisure time sitting group expending the most. In the present study, associations of physical activity and leisure time sitting combinations on risk of incident obesity were strongest after 5 years, with effects appearing weaker at a longer 10 year follow-up point. The dilution of effects over time might be due to misclassification errors resulting from changes in physical activity and leisure time sitting during the follow-up period [35] which were not possible to consider in the present study.

Our findings on incident metabolic risk factor clustering did not follow a pattern of additive interaction or dose–response. The greatest reduction in risk observed for intermediate levels of physical activity and leisure time sitting was unexpected and suggests that moderate amounts of both moderate-to-vigorous physical activity and leisure time sitting may be sufficient to protect against developing metabolic risk factor clustering over time. Results from previous studies on the interactive nature of physical activity and sitting with metabolic risk factor clustering are mixed. In some cross-sectional studies, higher sitting time was associated with metabolic risk factor clustering independently of physical activity [16, 36], while other studies suggest that the strength of the association between sitting and metabolic risk depends upon engagement in physical activity [37, 38]. Some prospective studies have suggested that higher objectively measured sitting time is associated with worsening insulin profiles [39] and with metabolic risk factor clustering [24] independently of moderate-to-vigorous physical activity, while others reported that increased television viewing time [40] and low physical activity both independently predicted worsening metabolic profiles after several years of follow-up.

The combined effects of physical activity and leisure time sitting on developing metabolic risk factor clustering have not been previously examined; however, results of present analyses seem discordant with expected patterns, given known dose–response associations of moderate-to-vigorous physical activity on risk of metabolic risk factor clustering and related diseases [8, 21, 22]. The U-shaped pattern of results presently observed may be due to chance or to residual confounding. Obese individuals at baseline were included in analyses of incident metabolic risk factor clustering. The proportion of participants with intermediate levels of physical activity and sitting who were obese was, however, greater than those with high activity and low sitting (Table 1); thus, confounding by BMI is unlikely. Antihypertensive and diabetic drug use formed part of the criteria for metabolic risk factor clustering; however, data on lipid-lowering drug use were not considered. Wider use of such medication by participants in intermediate groups could help explain their reduced risk of metabolic risk factor clustering. Another possible explanation involves misclassification errors resulting from changes in physical activity and leisure time sitting over time. For instance, participants reporting high physical activity and low leisure time sitting at baseline may have worsened their activity profile after this assessment, thus making their risk of metabolic risk factor clustering comparable to that of inactive individuals. ‘Metabolic risk factor clustering’ was treated as a binary outcome in the main analyses, the results of which may depend upon the specific cut-points chosen. To examine this possibility, we performed sensitivity analyses investigating associations of physical activity and leisure time sitting with change in the number of metabolic risk factors over time, with obesity included as one of six factors of interest. Results of these analyses fail to support physical activity and leisure time sitting as factors involved in the accumulation of metabolic risk factors. Strong effects were therefore observed for becoming obese when obesity was considered on its own, but not when grouped as one of several components of metabolic risk factor clustering. This finding is consistent with main analyses suggesting weaker results for metabolic risk factor clustering compared with the obesity outcome.

Associations of sedentary behaviour with metabolic risk may depend on the measure which is employed. For instance, associations are often weak or non-existent when using sitting in an occupational context as a marker of sedentary time [41, 42], while detrimental associations with abnormal glucose metabolism [43], insulin resistance, dyslipidaemia [44] and metabolic clustering [17, 37, 44] are widely reported when using self-reported television viewing as an indicator of sitting. We used a measure of total leisure time sitting in the present study, which may provide better insight into overall effects of sitting on adverse metabolic change than measures which are context-specific. In a related sense, the modern paradigm of sedentary behaviour as an independent health risk is based largely on observations that higher levels of sitting remain associated with metabolic risk factors after statistically accounting for engagement in moderate-to-vigorous activity [45]. However, independent associations of sitting on metabolic risk are less evident when adjusting for broader incidental measures of light intensity physical activity. For instance, when adjusting for total activity as objectively measured by accelerometry (and not moderate or vigorous activity only), associations between total sitting time and metabolic risk factors, including inflammatory markers and blood lipids, were no longer evident [46]. Nevertheless, light physical activity and sitting are highly correlated and are thus difficult to model together.

### Strengths and limitations

Main strengths of this study include a large sample size, a prospective design with follow-up extending to 10 years, and objective measures of anthropometrics and metabolic risk factors. We had the advantage of assessing incident obesity and incident metabolic risk factor clustering in the same study, thus affording direct comparisons between the two outcomes. Physical activity and leisure time sitting were measured on a continuous scale, allowing the use of percentile groups to better examine dose–response associations. Metabolic risk factor clustering was defined in this study according to comprehensive criteria used in previous work on the general US population [2]; however, C-reactive protein was not included as part of our definition as it was not available at the 10 year follow-up. Diet quality was assessed via frequency of fruit and vegetable consumption and may be subject to residual confounding by other aspects of diet such as excessive fat or sugar intake [47]. Measures of physical activity and leisure time sitting were taken at baseline only and are thus subject to misclassification errors if these are unstable over the course of follow-up [35]. Physical activity and leisure time sitting were self-reported and thus subject to biases. In particular, self-reported sitting time tends to be only moderately correlated with objective assessments [48]. However, given that subjective measures of physical activity and sitting have shown weaker and less consistent associations with metabolic risk factors compared with objective measures [48, 49], associations observed in present analyses may be, if anything, underestimates of true effects. Longitudinal studies using objective measures of physical activity and leisure time sitting combinations are needed to confirm these findings.

### Conclusions

The protective effects of moderate-to-vigorous physical activity and low leisure time sitting against developing obesity and metabolic risk factor clustering are strongest when viewed in combination. The interaction observed supports the notion that both high levels of physical activity and low levels of leisure time sitting may be required to substantially reduce the risk of developing obesity. Associations with developing metabolic risk factor clustering were less clear. Intervention studies are needed to examine whether adverse metabolic changes in the form of obesity and metabolic risk factor clustering are best prevented by improving levels of both physical activity and leisure time sitting.

## Electronic supplementary material

Below is the link to the electronic supplementary material.

ESM Table 1 (PDF 91 kb)

ESM Table 2 (PDF 104 kb)

ESM Table 3 (PDF 167 kb)

ESM Table 4 (PDF 105 kb)

## Abbreviations

HOMA-IR: HOMA of insulin resistance
MET: Metabolic equivalent
