# Supplementary material for: Combined effect of physical activity and leisure time sitting on long-term risk of incident obesity and metabolic risk factor clustering
Source: Diabetologia. 2014 Jul 31;57(10):2048–56. doi: 10.1007/s00125-014-3323-8 (PMC4153972; doi:10.1007/s00125-014-3323-8)
Supplement: Supplementary file 1 — (PDF 91 kb) [file 125_2014_3323_MOESM1_ESM.pdf]

ESM Table 1: Minimally adjusted separate associations of physical activity and leisure-time sitting level at baseline with incident obesity and incident metabolic risk factor clustering at follow-up

	**Incident obesity** Odds ratio (95% CI)		**Incident metabolic risk factor clustering** Odds ratio (95% CI)	
	**After 5 years** n=3160	**After 10 years** n=2778	**After 5 years** n=2529	**After 10 years** n=2254
Physical activity level				
Low	1.00 (Reference)	1.00 (Reference)	1.00 (Reference)	1.00 (Reference)
Intermediate	0.71 (0.50, 1.01)	0.63 (0.45, 0.87)	0.76 (0.60, 0.97)	0.75 (0.60, 0.94)
High	0.64 (0.44, 0.93)	0.63 (0.45, 0.88)	0.86 (0.67, 1.09)	0.83 (0.66, 1.05)
P-trend	*0.02*	*0.01*	*0.25*	*0.14*
Leisure-time sitting level				
High	1.00 (Reference)	1.00 (Reference)	1.00 (Reference)	1.00 (Reference)
Intermediate	0.78 (0.55, 1.11)	0.93 (0.67, 1.28)	0.77 (0.62, 0.97)	0.76 (0.61, 0.94)
Low	0.99 (0.69, 1.42)	1.08 (0.77, 1.51)	0.79 (0.62, 1.01)	0.78 (0.62, 0.99)
P-trend	*0.87*	*0.74*	*0.04*	*0.02*

Separate associations are mutually adjusted. Models adjusted for age, sex, and ethnicity.
