# Supplementary material for: Combined effect of physical activity and leisure time sitting on long-term risk of incident obesity and metabolic risk factor clustering
Source: Diabetologia. 2014 Jul 31;57(10):2048–56. doi: 10.1007/s00125-014-3323-8 (PMC4153972; doi:10.1007/s00125-014-3323-8)
Supplement: Supplementary file 2 — (PDF 104 kb) [file 125_2014_3323_MOESM2_ESM.pdf]

**ESM Table 2:** Minimally adjusted combined associations of physical activity and leisure-time sitting level at baseline with incident obesity and incident metabolic risk factor clustering at follow-up

**Incident obesity**

Odds ratio (95% CI)

| | After 5 years n=3160 | | | | After 10 years n=2778 | | | |
|---|---|---|---|---|---|---|---|---|
| | ***Leisure-time sitting level*** | | | | ***Leisure-time sitting level*** | | | |
| ***Physical activity level*** | *High* | *Intermediate* | *Low* | *P-trend* | *High* | *Intermediate* | *Low* | *P-trend* |
| *Low* | 1.00 (Reference) | 0.62 (0.35, 1.11) | 1.12 (0.65, 1.93) | *0.64* | 1.00 (Reference) | 0.88 (0.53, 1.47) | 1.30 (0.78, 2.17) | *0.26* |
| *Intermediate* | 0.53 (0.29, 0.97) | 0.52 (0.29, 0.95) | 0.89 (0.52, 1.53) | *0.10* | 0.56 (0.32, 1.00) | 0.63 (0.37, 1.08) | 0.77 (0.45, 1.32) | *0.42* |
| *High* | 0.76 (0.43, 1.32) | 0.63 (0.35, 1.12) | 0.23 (0.10, 0.57) | *0.01* | 0.78 (0.46, 1.32) | 0.66 (0.39, 1.13) | 0.47 (0.24, 0.91) | *0.15* |
| *P-trend* | *0.29* | *0.86* | *0.001* | | *0.28* | *0.41* | *0.003* | |
| *P-interaction* | | *0.02* | | | | *0.34* | | |

**Incident metabolic risk factor clustering**

Odds ratio (95% CI)

| | After 5 years n=2529 | | | | After 10 years n=2254 | | | |
|---|---|---|---|---|---|---|---|---|
| | ***Leisure-time sitting level*** | | | | ***Leisure-time sitting level*** | | | |
| ***Physical activity level*** | *High* | *Intermediate* | *Low* | *P-trend* | *High* | *Intermediate* | *Low* | *P-trend* |
| *Low* | 1.00 (Reference) | 0.63 (0.42, 0.93) | 0.71 (0.47, 1.09) | *0.10* | 1.00 (Reference) | 0.67 (0.46, 0.98) | 0.80 (0.53, 1.20) | *0.21* |
| *Intermediate* | 0.74 (0.51, 1.09) | 0.52 (0.35, 0.78) | 0.50 (0.33, 0.76) | *0.05* | 0.80 (0.55, 1.15) | 0.52 (0.36, 0.76) | 0.53 (0.36, 0.78) | *0.03* |
| *High* | 0.67 (0.46, 0.97) | 0.67 (0.46, 0.98) | 0.67 (0.45, 1.01) | *0.99* | 0.71 (0.50, 1.03) | 0.67 (0.47, 0.97) | 0.64 (0.43, 0.95) | *0.56* |
| *P-trend* | *0.01* | *0.74* | *0.48* | | *0.07* | *0.90* | *0.65* | |
| *P-interaction* | | *0.41* | | | | *0.56* | | |

Models adjusted for age, sex, and ethnicity.
