# Supplementary material for: Combined effect of physical activity and leisure time sitting on long-term risk of incident obesity and metabolic risk factor clustering
Source: Diabetologia. 2014 Jul 31;57(10):2048–56. doi: 10.1007/s00125-014-3323-8 (PMC4153972; doi:10.1007/s00125-014-3323-8)
Supplement: Supplementary file 3 — (PDF 167 kb) [file 125_2014_3323_MOESM3_ESM.pdf]

**ESM Table 3:** Separate associations of physical activity and leisure-time sitting level at baseline with change in number of metabolic risk factors at follow-up compared with baseline

| | **Change in number of metabolic risk factors** Unstandardized B (95% CI) | |
|---|---|---|
| | **After 5 years** n=3670 | **After 10 years** n=3214 |
| | **Model 1** | |
| ***Physical activity level*** | | |
| *Low* | 0.00 (Reference) | 0.00 (Reference) |
| *Intermediate* | -0.05 (-0.14, 0.04) | 0.04 (-0.06, 0.14) |
| *High* | -0.01 (-0.10, 0.08) | 0.07 (-0.04, 0.17) |
| *P-trend* | *0.86* | *0.21* |
| ***Leisure-time sitting level*** | | |
| *High* | 0.00 (Reference) | 0.00 (Reference) |
| *Intermediate* | -0.06 (-0.14, 0.03) | 0.01 (-0.08, 0.11) |
| *Low* | -0.02 (-0.11, 0.07) | 0.06 (-0.04, 0.16) |
| *P-trend* | *0.59* | *0.25* |
| | **Model 2** | |
| ***Physical activity level*** | | |
| *Low* | 0.00 (Reference) | 0.00 (Reference) |
| *Intermediate* | -0.04 (-0.13, 0.05) | 0.04 (-0.06, 0.14) |
| *High* | -0.00 (-0.10, 0.09) | 0.08 (-0.03, 0.18) |
| *P-trend* | *0.96* | *0.14* |
| ***Leisure-time sitting level*** | | |
| *High* | 0.00 (Reference) | 0.00 (Reference) |
| *Intermediate* | -0.06 (-0.14, 0.03) | 0.02 (-0.08, 0.11) |
| *Low* | -0.01 (-0.10, 0.08) | 0.07 (-0.04, 0.17) |
| *P-trend* | *0.68* | *0.21* |

Separate associations are mutually adjusted. **Model 1** adjusted for age, sex, and ethnicity. **Model 2** further adjusted for occupational position, frequency of fruit and vegetable consumption, smoking status, alcohol consumption, and the presence of a long-standing illness.
