# Supplementary material for: Combined effect of physical activity and leisure time sitting on long-term risk of incident obesity and metabolic risk factor clustering
Source: Diabetologia. 2014 Jul 31;57(10):2048–56. doi: 10.1007/s00125-014-3323-8 (PMC4153972; doi:10.1007/s00125-014-3323-8)
Supplement: Supplementary file 4 — (PDF 105 kb) [file 125_2014_3323_MOESM4_ESM.pdf]

ESM Table 4: Combined associations of physical activity and leisure-time sitting level at baseline with incident obesity and incident metabolic risk factor clustering at follow-up

	**Change in number of metabolic risk factors** Unstandardized B (95% CI)							
	**After 5 years** n=3670				**After 10 years** n=3214			
**Model 1**								
	Leisure-time sitting level				***Leisure-time sitting level***			
Physical activity level	*High*	*Intermediate*	*Low*	*P-trend*	*High*	*Intermediate*	*Low*	*P-trend*
Low	0.00 (Reference)	-0.12 (-0.27, 0.02)	0.01 (-0.15, 0.17)	*0.93*	0.00 (Reference)	0.02 (-0.15, 0.18)	0.04 (-0.13, 0.22)	*0.60*
Intermediate	-0.08 (-0.23, 0.06)	-0.08 (-0.22, 0.06)	-0.11 (-0.26, 0.04)	*0.67*	0.08 (-0.08, 0.25)	0.01 (-0.15, 0.17)	0.09 (-0.08, 0.26)	*0.98*
High	-0.02 (-0.16, 0.12)	-0.08 (-0.22, 0.07)	-0.05 (-0.21, 0.11)	*0.65*	0.02 (-0.14, 0.18)	0.10 (-0.06, 0.26)	0.15 (-0.03, 0.33)	*0.11*
P-trend	*0.44*	*0.74*	*0.90*		*0.85*	*0.33*	*0.28*	
P-interaction		*0.78*				*0.81*		
**Model 2**								
	Leisure-time sitting level				***Leisure-time sitting level***			
Physical activity level	*High*	*Intermediate*	*Low*	*P-trend*	*High*	*Intermediate*	*Low*	*P-trend*
Low	0.00 (Reference)	-0.12 (-0.26, 0.03)	0.01 (-0.14, 0.17)	*0.99*	0.00 (Reference)	0.03 (-0.13, 0.20)	0.05 (-0.13, 0.23)	*0.54*
Intermediate	-0.08 (-0.22, 0.07)	-0.08 (-0.22, 0.07)	-0.10 (-0.25, 0.05)	*0.81*	0.10 (-0.07, 0.26)	0.02 (-0.14, 0.18)	0.11 (-0.06, 0.28)	*0.94*
High	-0.02 (-0.16, 0.13)	-0.07 (-0.21, 0.08)	-0.04 (-0.20, 0.12)	*0.74*	0.04 (-0.13, 0.20)	0.13 (-0.04, 0.29)	0.17 (-0.01, 0.35)	*0.10*
P-trend	*0.33*	*0.56*	*0.57*		*0.90*	*0.35*	*0.08*	
P-interaction		*0.46*				*0.47*		

Model 1 adjusted for age, sex, and ethnicity. **Model 2** further adjusted for occupational position, frequency of fruit and vegetable consumption, smoking status, alcohol consumption, and the presence of a long-standing illness.
